# Serum miR-339-3p as a potential diagnostic marker for non-small cell lung cancer

**DOI:** 10.20892/j.issn.2095-3941.2020.0063

**Published:** 2020-08-15

**Authors:** Keson Trakunram, Pichitpon Chaniad, Sarayut Lucien Geater, Warangkana Keeratichananont, Voravit Chittithavorn, Sumonmal Uttayamakul, Suhaimee Buya, Pritsana Raungrut, Paramee Thongsuksai

**Affiliations:** ^1^Department of Biomedical Sciences, Faculty of Medicine, Prince of Songkla University, Hat Yai, Songkhla 90110, Thailand; ^2^Department of Internal Medicine, Faculty of Medicine, Prince of Songkla University, Hat Yai, Songkhla 90110, Thailand; ^3^Department of Surgery, Faculty of Medicine, Prince of Songkla University, Hat Yai, Songkhla 90110, Thailand; ^4^Bamrasnaradura Infectious Diseases Institute, Nonthaburi 11000, Thailand; ^5^Medical Data Center for Research and Innovation, Faculty of Medicine, Prince of Songkla University, Hat Yai, Songkhla 90110, Thailand; ^6^Department of Pathology, Faculty of Medicine, Prince of Songkla University, Hat Yai, Songkhla 90110, Thailand

**Keywords:** Biomarker, diagnosis, MicroRNA, non-small cell lung cancer, quantitative real-time polymerase chain reaction

## Abstract

**Objective**: MicroRNA (miRNA), a short noncoding RNA, is claimed to be a potential blood-based biomarker. We aimed to identify and evaluate miRNAs as diagnostic biomarkers for non-small cell lung cancer (NSCLC).

**Methods**: Profiles of 745 miRNAs were screened in the serum of 8 patients with NSCLC and 8 age- and sex-matched controls using TaqMan low-density arrays (TLDAs) and validated in 25 patients with NSCLC and 30 with other lung diseases (OLs) as well as in 19 healthy persons (HPs). The diagnostic performance of the candidate miRNAs was assessed in 117 cases of NSCLC and 113 OLs using quantitative real-time polymerase chain reaction (qRT-PCR). Differences in miRNA expression between patients with NSCLC and controls were assessed using the Mann–Whitney U test. The area under receiver operating characteristic (ROC) curve (AUC) was obtained based on the logistic regression model.

**Results**: Ten miRNAs were found to be differentially expressed between patients with NSCLC and controls, including miR-769, miR-339-3p, miR-339-5p, miR-519a, miR-1238, miR-99a#, miR-134, miR-604, miR-539, and miR-342. The expression of miR-339-3p was significantly higher in patients with NSCLC than in those with OLs (*P* < 0.001) and HPs (*P* = 0.020). ROC analysis revealed an miR-339-3p expression AUC of 0.616 [95% confidence interval (CI): 0.561–0.702]. The diagnostic prediction was increased (AUC = 0.706, 95% CI: 0.649–0.779) in the model combining miR-339-3p expression and other known risk factors (i.e., age, smoking status, and drinking status).

**Conclusions**: MiR-339-3p was significantly upregulated in patients with NSCLC compared with participants without cancer, suggesting a diagnostic prediction value for high-risk individuals. Therefore, miR-339-3p expression could be a potential blood-based biomarker for NSCLC.

## Introduction

Lung cancer is one of the most frequently diagnosed cancers and is the leading cause of cancer deaths worldwide. In 2018, it contributed to 11.6% of all cancer cases and 18.4% of cancer deaths^1^. Histologically, non-small cell lung cancer (NSCLC) accounts for 80%–85% and small-cell lung cancer accounts for 10%–15% of all cases of lung cancer^2^. The high mortality rate of lung cancer is primarily related to the stage of disease before treatment. The 5-year survival rate of stage I lung cancer is 56%, which reduces to 5% in patients with stage IV cancer^2^. Low-dose computed tomography^3^, a recommended screening method, is of limited value owing to concerns such as radiation exposure, cost-effectiveness, and applicability in a clinical setting^3,4^. Likewise, tissue biopsy using bronchoscopy, a standard conventional method, is an invasive procedure with its associated risks. Therefore, searching for a less-invasive diagnostic method that is easily accessible in a clinical setting is an important focus of current research.

MicroRNAs (miRNAs) are single-stranded noncoding small RNA molecules measuring 19–22 nucleotides in length. They regulate the post-transcriptional expression of target mRNAs by binding to the 3′-untranslated region (3′-UTR), leading to the repression of associated genes^5^. Previous studies have demonstrated that miRNAs play crucial roles in tumorigenesis by acting as oncogenes or tumor-suppressor genes^6,7^. Given miRNAs have been shown to possess high stability in various biological samples, including plasma and serum, considerable interest has arisen regarding their potential role as blood-based biomarkers. Several studies have shown that miRNAs are promising blood-based biomarkers for use in diagnosis, prognosis, and prediction of treatment success in various types of cancer^8–11^.

Studies on miRNA profiling in lung cancer have been conducted^12–15^. However, the list of differentially expressed miRNAs in these studies is inconsistent. This variation could be because of different profiling platforms and/or different characteristics of the study populations. In addition, many studies employed healthy controls as non-cancer cases in their diagnostic evaluation; this approach has been criticized owing to the possibility of inflated results^16^.

In the present study, we aimed to identify miRNAs that might be potential diagnostic NSCLC markers through miRNA profiling using TaqMan low-density arrays (TLDAs). The differentially expressed miRNAs were validated in an independent set of samples using quantitative real-time polymerase chain reaction (qRT-PCR). We included healthy individuals as well as patients with other lung diseases (OLs) as controls. We then assessed the diagnostic performance of the candidate miRNA in a series of patients who had symptoms suspected to indicate lung cancer.

## Materials and methods

### Patients and controls

This prospective study was approved by the Human Research Ethics Committee of the Faculty of Medicine, Prince of Songkla University (REC: 59-011-05-1 and REC: 60-350-04-2). The study participants were selected from the patient population at Songklanagarind Hospital, Songkhla, Thailand between 2016 and 2018. All blood samples were collected from participants after informed consent was obtained.

**Figure 1:**
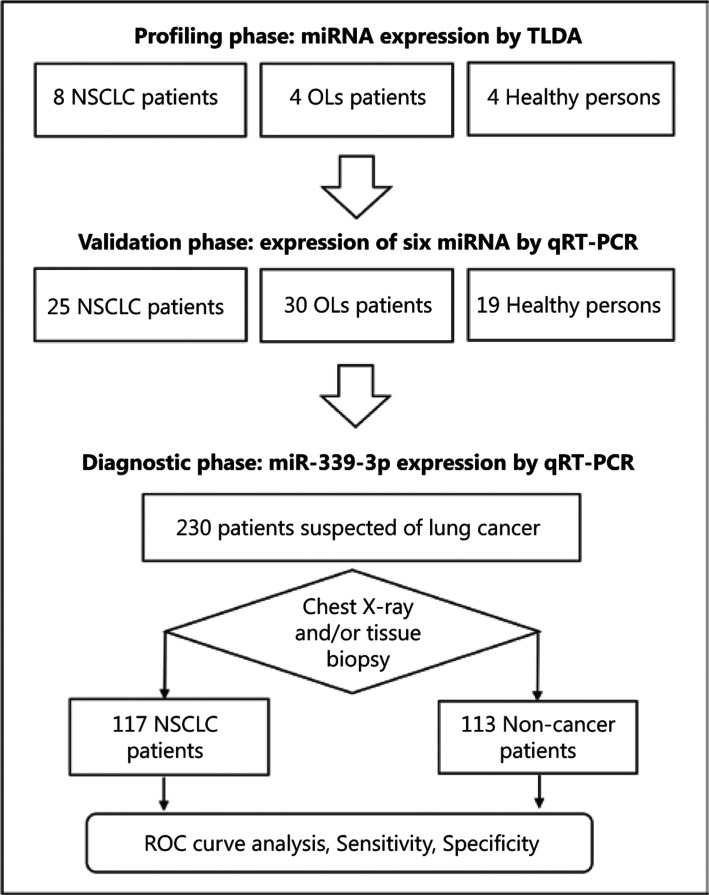
Overall study design and number of participants in each phase of the study. NSCLC, non-small cell lung cancer; OLs, other lung diseases.

The overall study design and number of participants are shown in **Figure 1**. The study was divided into three phases: profiling, validation, and diagnostic evaluation. In the first two phases, the participants were enrolled in a case–control manner. Patients included those who were newly diagnosed with NSCLC of any stage, whereas the controls were patients with OLs and healthy persons (HPs) who were age- (±5 years) and sex-matched with the patients with NSCLC. OL was diagnosed using some or all of the following methods: clinical history, laboratory findings, chest X-ray, and/or tissue biopsy. HPs with normal chest X-rays were recruited at a check-up clinic. None of the controls had been previously diagnosed with cancer.

A cohort design was used for the diagnostic evaluation phase. Patients who had chronic cough for at least 8 weeks or hemoptysis more than once were recruited. Cancer diagnosis was confirmed by tissue biopsy in all cases. Histological diagnoses were achieved according to the 2015 World Health Organization classification for lung and pleural tumors^17^. Chest X-ray and/or clinical follow-up for at least 6 months was applied in cases for which biopsy was not indicated. Patients with asthma, gastroesophageal reflux disease, allergic rhinitis, and acute pneumonia were excluded.

Demographic and clinical data were obtained from medical records. History-taking regarding smoking and drinking habits and family history of cancer was performed through interviews using a structured questionnaire. Patients who were diagnosed before January 2018 were clinically staged based on the seventh edition^18^ of the American Joint Committee on Cancer’s Cancer Staging Manual and those diagnosed from January 2018 onward were staged according to the eighth edition^19^. Blood samples were obtained from the patients with NSCLC before they received any cancer treatment.

### Blood collection

Whole-blood sample (5 mL) was collected from each participant and placed in a clotting tube (Greiner Bio-One, Kremsmünster, Austria) and kept at room temperature for no longer than 30 min. The blood samples were centrifuged at 3400 *g* for 10 min at room temperature, and the supernatant was then filtrated through a polyvinylidene difluoride syringe with a 0.22-µm pore (Merck Millipore, Darmstadt, Germany). Aliquoted serum samples were stored at −80 °C until use.

### RNA extraction

Total RNA was extracted from 250 µL serum using an miRNeasy mini kit (Qiagen, Hilden, Germany) according to the manufacturer’s protocol. Before RNA extraction, synthetic cel-miR-39 RNA (5′-UCACCGGGUGUAAAUCAGCUUG-3′) at a final concentration of 1.6 × 10^8^ copies/µL was added to each sample to assess extraction efficacy. The quantity and quality (OD 260/280 ratio and 260/230 ratio, respectively) of total RNA were determined using a NanoDrop ND-1000 UV-Vis spectrophotometer (Thermo Fisher Scientific, Waltham, MA, USA).

### MiRNA profiling

The expression profiling of 745 miRNAs was performed using TLDAs (TLDA version 3.0 cards A and B; Life Technologies, Carlsbad, CA, USA) according to the manufacturer’s instructions. Briefly, 100 ng of total RNA was reverse transcribed to complementary DNA (cDNA) using a TaqMan miRNA reverse transcription (RT) kit and stem-loop Megaplex RT primers (human pools A and B). RT conditions were set as follows: 40 cycles of 16 °C for 2 min, 42 °C for 1 min, and 50 °C for 1 s, and 85 °C for 5 min, with subsequent holding at 4 °C in a 7500 RT-PCR system (Life Technologies). To improve the sensitivity, the cDNA was preamplified using Megaplex PreAmp primers and TaqMan PreAmp Master Mix. The conditions for preamplification were set as follows: 95 °C for 10 min, 55 °C for 2 min, 75 °C for 2 min, 12 cycles of 95 °C for 15 s, and 60 °C for 4 min, with subsequent holding at 4 °C in a 7500 Fast RT-PCR system (Life Technologies).

The preamplified product was diluted with 75 µL of ribonuclease-free water and mixed with 2× TaqMan Universal PCR Master Mix without AmpErase UNG. Expression profiling was performed using a QuantStudio^TM^ 7 Flex RT-PCR system (Life Technologies) for 50 cycles of 30 min at 16 °C, 30 min at 42 °C, and 5 min at 85 °C, with subsequent holding at 4 °C. Small nuclear RNA6 (RNU6) was used as an internal control.

The raw cycle threshold (Ct) values were exported to the ExpressionSuite version 1.0.4 software (Thermo Fisher Scientific) to set baseline and threshold values. miRNAs with a Ct value less than the threshold value or with no signal were defined as undetected miRNAs. In these cases, the Ct value was set at 50 (the maximum cycle set in this experiment) for further analysis. Relative miRNA expression levels were calculated using the equation: ∆Ct = CtmiRNA − CtRNU6. Differential expression values between the patients with NSCLC and controls were obtained using the equation: ∆∆Ct = (CtmiRNA − CtRNU6) NSCLC − (CtmiRNA − CtRNU6) control. The fold change of the relative expression of miRNAs was calculated using the 2^−(∆∆Ct)^ method^20^.

### MiRNA expression by qRT-PCR

For validation and diagnostic evaluation, the expression level of each miRNA was determined using qRT-PCR. TRIzol^®^LS reagent (Invitrogen, Carlsbad, CA, USA) was used to extract total RNA from the serum. Briefly, 600 µL of TRIzol^®^LS reagent was added to 200 µL of serum, mixed together by inversion, and incubated for 15 min at room temperature. Then, a synthetic cel-miR-39 was added to each sample as an external control. After adding 200 µL of chloroform (J.T. Baker, Center Valley, PA, USA), the mixture was inverted for 15 s and allowed to stand for 5 min at room temperature. Following centrifugation at 12,000 *g* at 4 °C for 15 min, the total RNA was precipitated at the aqueous phase by adding 500 µL of isopropanol (J.T. Baker) and then resuspended in 20 µL of ribonuclease-free water (Qiagen). The quantity and quality of total RNA were assessed using a NanoDrop^®^ND-1000 UV-Vis spectrophotometer.

To synthesize cDNA, 50 ng of the total RNA was subjected to RT reaction using an miScript RT kit (Qiagen) according to the manufacturer’s instructions. The RT reaction was incubated at 37 °C for 60 min and then inactivated by heating at 95 °C for 5 min using a thermal cycler (Bio-Rad Laboratories, Hercules, CA, USA). qRT-PCR was performed using a BioRad CFX96 qPCR system (Bio-Rad) with a miScript SYBR^®^Green PCR kit (Qiagen). An miScript primer assay (Qiagen) was used for universal primer, and mature miRNA sequences were used for designing the following forward primers: miR-769, UGAGACCUCUGGGUUCUGAGCU; miR-339-3p, UGAGCGCCUCGACGACAGAGCCG; miR-339-5p, UCCCUGUCCUCCAGGAGCUCACG; miR-134, UGUGACUGGUUGACCAGAGGGG; miR-342, AGGGGUGCUAUCUGUGAUUGA; and miR-604, AGGCUGCGGAAUUCAGGAC. The qPCR amplification conditions were as follows: 95 °C for 15 min, followed by 50 cycles of 94 °C for 15 s; 55 °C (cel-miR-39, RNU6, miR-769, miR-339-3p, miR-604, miR-342, and miR-134) or 62.5 °C (miR-339-5p) for 30 s; and 70 °C for 30 s. The 2^−(ΔCt)^ method was used to calculate the relative expression levels of each miRNA^20^.

### Bioinformatics analysis

An online database, miRTarBase (http://mirtarbase.mbc.nctu.edu.tw/php/index.php), was consulted to determine the miRNA-target genes. These target genes have been functionally validated by a variety of robust methods, such as reporter assay, western blotting, and qRT-PCR, as well as by less-robust approaches including microarray and next-generation sequencing. Pathway enrichment analyses of the target genes were determined using the Kyoto Encyclopedia of Genes and Genomes *via* the Database for Annotation, Visualization, and Integrated Discovery (https://david.ncifcrf.gov/).

### Statistical analysis

The distribution of demographic and clinical characteristics among the study groups was described as frequency (percentage) or mean [±standard deviation (SD)] as appropriate. The differences in these variables between the patients with lung cancer and those without lung cancer were assessed using the chi-squared test or Fisher’s exact test for categorical variables and using the *t*-test or Mann–Whitney U test (non-normally distributed data) for quantitative variables as appropriate. The relative expression value of miRNA (2^(−∆Ct)^) was naturally log-transformed for additional analyses. Differences in miRNA levels across the three groups of participants were examined by one-way analysis of variance and between the two groups using the *t*-test. Receiver operating characteristic (ROC) curves were generated, and the corresponding areas under the curve (AUCs) were obtained based on the logistic regression model. *P*-value ≤ 0.050 was considered statistically significant. R software was used for statistical analyses. Visualization of microarray data as a heat map was performed using the ClustVis tool (https://biit.cs.ut.ee/clustvis/). GraphPad Prism version 5.0 (Graphpad Software Inc., San Diego, CA, USA) was used to create box plots.

## Results

Clinical characteristics of participants

The numbers of participants involved in each phase of the study are shown in **Figure 1**. The participants in the profiling set included 8 patients with NSCLC, 4 with OLs (2 patients with tuberculosis and 2 with chronic obstructive pulmonary disease), and 4 HPs. The validation set included 25 patients with NSCLC, 19 HPs, and 30 patients with OLs, which included those with bronchitis (*n* = 20), bronchiectasis (*n* = 6), tuberculosis (*n* = 2), pneumonia (*n* = 1), and idiopathic pulmonary fibrosis (*n* = 1). The diagnostic set comprised patients who presented with chronic cough or hemoptysis including 117 patients with NSCLC and 113 with OLs. The specific diagnoses of OLs were bronchitis (*n* = 55), bronchiectasis (*n* = 16), pneumonia (*n* = 16), tuberculosis (*n* = 25**)**, and pulmonary fibrosis (*n* = 1). **Table 1** summarizes the demographic and clinical characteristics of all participants involved in the three phases of our study. The demographic and lifestyle habits of patients with NSCLC and those without cancer were not different except for smoking status in the diagnostic set (i.e., there were 20% more smokers among lung cancer cases). The majority of patients with NSCLC had advanced-stage disease.

### Serum miRNA profiling and differential expression

**Figure 2:**
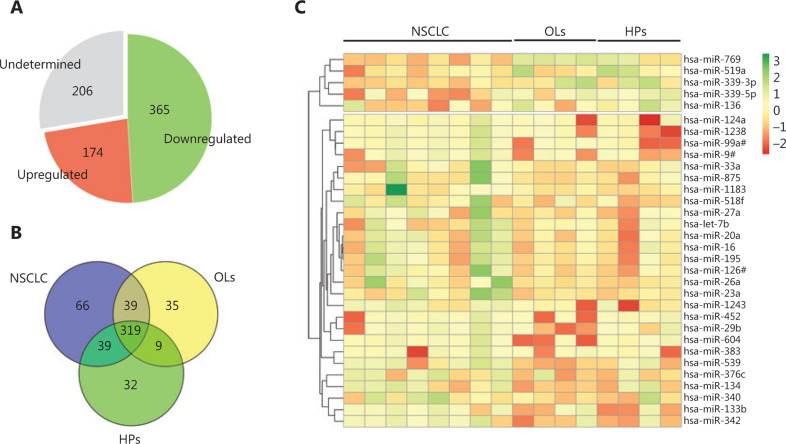
Expression profiles of miRNAs. (A) Pie chart depicting the number of undetected, upregulated, and downregulated miRNAs. (B) Venn diagram of detected miRNAs in three groups of samples. (C) Heat map of differentially expressed miRNAs (*P* < 0.6). Red indicates high-expression miRNAs and green indicates low-expression miRNAs. HPs, healthy persons; NSCLC, non-small cell lung cancer; OLs, other lung diseases.

A total of 539 miRNAs were detected, of which 174 were upregulated and 365 were downregulated (**Figure 2A**). Furthermore, 66, 35, and 32 miRNAs were detected in the NSCLC, OL, and HP groups, respectively (**Figure 2B**). Hierarchical cluster analysis showed two distinct miRNA expression patterns between the NSCLC and control groups (**Figure 2C**). Ten miRNAs were found to be differentially expressed (*P* < 0.050) between the patients with NSCLC and all controls or between the patients with NSCLC and each control group (**Table 2**). These included miR-769, miR-339-3p, miR-339-5p, miR-519a, miR-1238, miR-99a#, miR-134, miR-604, miR-539, and miR-342.

### Validation of differentially expressed miRNAs by qRT-PCR

**Figure 3:**
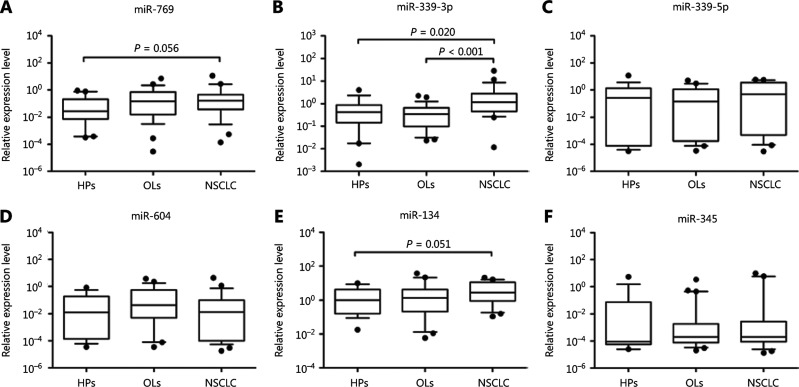
Box plots of the relative expression values of six miRNAs among three sample groups in a validation study using qRT-PCR. (A) miR-769, (B) miR-339-3p, (C) miR-339-5p, (D) miR-604, (E) miR-134, and (F) miR-342. HPs, healthy persons; NSCLC, non-small cell lung cancer; OLs, other lung diseases.

We selected 6 miRNAs (miR-769, miR-339-3p, miR-339-5p, miR-604, miR-134, and miR-342) that were significantly different between NSCLC patients versus all controls or between NSCLC versus OL patients. Validation was performed in 25 patients with NSCLC, 30 with OLs, and 19 HPs using qRT-PCR. The relative expressions of these miRNAs in each sample group are shown in **Figure 3**. The results revealed that miR-339-3p was significantly upregulated in patients with NSCLC compared with those with OLs (*P* < 0.001) and the HPs (*P* = 0.020), whereas miR-769 and miR-134 showed differential expressions between the NSCLC and HP groups with marginal significance (*P* = 0.056 and *P* = 0.051, respectively). The remaining miRNAs showed no significant differences in any of the comparisons.

### Target predictions and functional analysis of miR-339-3p using bioinformatics tools

In total, 14 miR-339-3p target genes were identified by the miRTarBase (**Table 3**). Three genes—namely, *MCL1*, *NFKB1*, and *FOXO1*—presented strong experimental evidence that supported the miRNA–target interaction. Enrichment analyses showed that the miR-339-3p target genes are involved in critical pathways related to cancer, such as the PI3K–Akt signaling pathway, AMPK signaling pathway, FoxO signaling pathway, and HIF-1 signaling pathway. However, 8 genes had no available pathway information.

### Diagnostic performance of serum miR-339-3p expression

**Figure 4:**
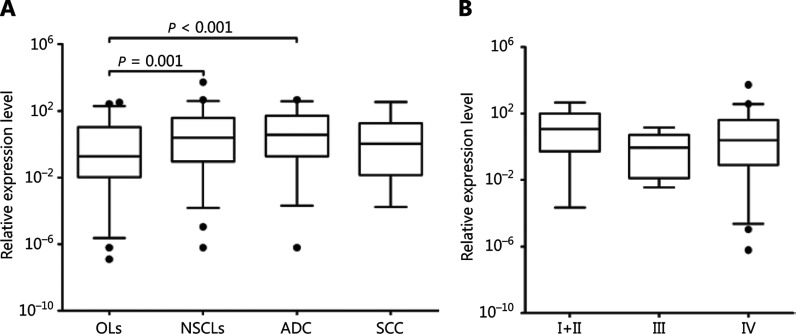
Box plots showing the log-transformed relative expression of serum miR-339-3p in a diagnostic study using qRT-PCR. (A) miR-339-3p expression in patients with OLs and different histologic types. (B) miR-339-3p in different clinical stages. ADC, adenocarcinoma; NSCLC, non-small cell lung cancer; OLs, other lung diseases; SCC, squamous cell carcinoma.

The serum expression of miR-339-3p was evaluated in 117 patients with NSCLC and 113 patients with OLs. Patients with NSCLC had significantly higher serum miR-339-3p expression (mean: 3.40, SD: 5.15) when compared with those with OLs (mean: 1.79, SD: 2.59) (*P* = 0.001). However, there was no significant difference in the miR-339-3p expression according to clinical parameters such as histologic type (*P* = 0.405) and clinical stage (*P* = 0.413) (**Figure 4**).

The diagnostic predictability of miR-339-3p expression with adjustment of other known risk factors was assessed by logistic regression. miR-339-3p was categorized into low (relative miR-339-3p expression < 0.593) and high expression. Age, smoking status, and miR-339-3p expression were significant predictors of lung cancer (**Table 4**). High expression of miR-339-3p significantly predicted lung cancer diagnosis [odds ratio (OR): 2.43, 95% confidence interval (CI): 1.39–4.25; *P* = 0.002]. Alcohol was found to confound the effect of smoking as the OR of smoking significantly changed when alcohol was present in the model. Therefore, we included drinking status in the final model. We also performed the analyses separately for both histologic types. The results of adenocarcinoma (ADC) are similar to those of the model of all histologic types, with a slightly stronger association of miR-339-3p (OR: 2.96, 95% CI: 1.57–5.58) (**Table 4**). miR-339-3p and squamous cell carcinoma (SCC) also showed a positive association (OR: 2.20, 95% CI: 0.86–5.67; *P* = 0.101), although it was not statistically significant. This is likely because of the small number of SCC patients (*n* = 22).

**Figure 5:**
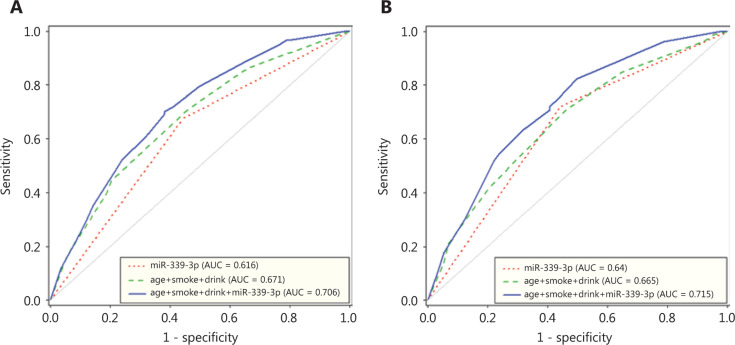
ROC analysis for the diagnosis of (A) NSCLC and (B) adenocarcinoma. ROC curves for miR-339-3p, clinical factors, and clinical factors with miR-339-3p are shown. NSCLC, non-small cell lung cancer.

We then generated ROC curves for different combinations of factors. For the prediction of NSCLC, the AUC of miR-339-3p alone was 0.616 (95% CI: 0.561–0.702), with a sensitivity of 68.5% and a specificity of 55.8 % at a relative miR-339-3p expression of 1.652. The AUC of clinical factors (i.e., age, smoking, and alcohol status) was 0.671 (95% CI: 0.612–0.745), whereas that of combined clinical factors and miR-339-3p increased to 0.706 (95% CI: 0.649–0.779) (**Figure 5A**). For the model involving ADC alone, the prediction was slightly better than that for all NSCLC patients. The AUC of miR-339-3p alone was 0.64 (95% CI: 0.572–0.707), whereas that of miR-339-3p combined with clinical factors was 0.715 (95% CI: 0.643–0.787) (**Figure 5B**).

## Discussion

We found a set of 10 differentially expressed miRNAs through miRNA profiling using the serum of patients with NSCLC and those without cancer. miR-339-3p was confirmed to be differentially expressed in the validation step. Further clinical evaluation showed that miR-339-3p expression was a fair diagnostic classifier (AUC: 0.616, 95% CI: 0.561–0.702). In addition, the diagnostic prediction was increased (AUC: 0.706, 95% CI: 0.649–0.779) in the model with combined miR-339-3p expression and other known risk factors.

In the profiling results, we found 10 differentially expressed miRNAs; among these, four miRNAs—miR-339-3p, miR-339-5p, miR-99a#, and miR-342—have been previously reported in other lung cancer profiling studies^13,21,22^. However, even though several miRNA profiling studies aiming to identify potential biomarkers for lung cancer diagnosis have been conducted, the lists of differentially expressed miRNAs mostly do not overlap. Certain factors contribute to this inconsistency—for example, the use of different profiling technologies. The platforms commonly used in miRNA profiling are microarray hybridization, qRT-PCR-based profiling, and massive parallel/next-generation sequencing^23^. Each platform has inherent strengths and limitations^24,25^. We used a qRT-PCR-based method, rather than hybridization or sequencing-based platforms, owing to its sensitivity, simplicity, and lower cost^23^. However, even when using the same profiling technology in the process, different reagent kits (for miRNA isolation in particular) could yield remarkably different numbers of detected miRNAs^26^.

We found that miR-339-3p expression was significantly higher in the patients with NSCLC than in the controls without cancer. The upregulation of miR-339-3p in NSCLC has also been reported in two previous studies^13,21^. Chen et al.^21^ profiled miRNA in 11 patients with NSCLC and 11 HPs using Solexa sequencing, whereas Nadal et al.^13^ performed miRNA profiling in 70 patients with NSCLC and 22 HP and OL controls using a TaqMan open-array human panel. Unfortunately, these authors did not include miR-339-3p in their validation studies. Increased expression of miR-339-3p has also been reported in other cancers; for example, it was shown to be upregulated in Hodgkin’s lymphoma tissue^27^ and in the plasma of patients with prostate cancer^28^. Therefore, miR-339-3p might function as an oncogenic miRNA. However, some studies have reported on the tumor-suppressor role of miR-339-3p. The overexpression of miR-339-3p inhibits the proliferation of colorectal cancer cells, and blocking miR-339-3p using anti-miRs has been suggested to increase melanoma cell invasion through direct interaction with *MCL1*^29^. Given miRNA can interact with a number of genes^30^, it can be speculated that miR-339-3p plays different roles depending on its gene targets. However, the biological mechanism of miR-339-3p in lung cancer tumorigenesis needs to be further explored.

In our diagnostic evaluation, miR-339-3p expression appeared to be a fair classifier for overall NSCLC (AUC: 0.616, 95% CI: 0.561–0.702) and ADC diagnosis (AUC: 0.64, 95% CI: 0.572–0.707). These values are lower than those in previous reports^9,31-34^. The discrepancy in the discrimination values depends on various factors. One of the important issues is the nature of participants without cancer in the diagnostic evaluation study. Many diagnostic studies apply a case–control design and include healthy individuals or patients with irrelevant conditions as a comparison group. This strategy is subject to a potential overestimation of test accuracy. Indeed, a typical diagnostic study is cross-sectional in nature, and in this context, a series of patients in the relevant clinical situation who also undergo the index test would be an ideal testing population^16^. In our study, we assessed the diagnostic performance of miR-339-3p expression in a series of patients who had clinical symptoms commonly found in lung cancer, i.e., prolonged cough and hemoptysis. This more or less contributed to the lower diagnostic performance in our study compared with that in other studies that used healthy controls or patients with irrelevant disorders^33–35^. Another reason for the relatively low AUC in our study is that only one miRNA was included in the model, whereas panels of up to 24 miRNAs were simultaneously tested in other studies^31,33^. However, incorporating many miRNAs will lead to higher costs in actual clinical practice. Therefore, determining the optimum biomarker panel that efficiently adds clinical diagnostic value in a clinical setting is challenging.

In the diagnostic scheme, biomarker testing serves as an additional test to distinguish individuals who have a set of certain symptoms or signs as to whether they are likely to have cancer. Therefore, incorporating clinical factors and known risk factors into the prediction model can improve diagnostic accuracy. In our study, we observed an increased AUC in the model having miR-339-3p combined with other known risk factors (i.e., age, smoking, and drinking) (AUC = 0.706, 95% CI: 0.649–0.779) compared with the miRNA model or with clinical factors alone. These results are consistent with those of other reports using a similar approach. For example, Wang et al.^32^ reported an AUC of 0.865 (95% CI: 0.821–0.902) for a model with 10 miRNAs and 6 symptoms compared with an AUC of 0.750 (95% CI: 0.697–0.798) for miRNAs alone. Wozniak et al.^31^ observed a higher AUC of 0.94 (95% CI: 0.90–0.97) for a model involving 24 miRNAs, sex, age, and smoking status compared with an AUC of 0.92 (95% CI: 0.87–0.95) for miRNAs alone. Therefore, the use of combined information of clinical symptoms and/or known risk factors as well as miRNAs should be applied for clinical practice.

There are limitations to the present study. The number of samples in the profiling set was small, which could affect the power of the study to detect the differences; furthermore, it might contribute to the small number of differentially expressed miRNAs in our study. In addition, owing to the small number of cases with SCC, the current study had limited power to evaluate the prediction capacity for this histologic type. Lastly, because the patients in our study mainly had advanced-stage cancer, this sample might not be the most suitable choice to search for early diagnostic biomarkers. Despite these limitations, we found one significant differentially expressed miRNA (miR-339-3p), which was technically and clinically validated with respect to its potential value as a diagnostic biomarker. To the best of our knowledge, miR-339-3p has never been reported previously as a potential biomarker in lung cancer.

In conclusion, our study demonstrated that miR-339-3p was significantly upregulated in patients with NSCLC and could be a potential diagnostic biomarker for this disease. In addition, a diagnostic prediction value is added for individuals suspected to have NSCLC. Therefore, it might be used as an adjunctive noninvasive laboratory test for these individuals. The biological role of miR-339-3p in lung cancer tumorigenesis requires further study to improve diagnostic performance.

## Figures and Tables

**Table 1:** Demographic and clinical characteristics of all participants in the three phases of the study

Variables	Profiling phase (No., %)		Validation phase (No., %)		Diagnostic phase (No., %)	
	NSCLC (*n* = 8)	OLs & HPs (*n* = 8)	NSCLC (*n* = 25)	OLs & HPs (*n* = 49)	NSCLC (*n* = 117)	OLs (*n* = 113)
Age (Mean±SD)	66.7±2.3	66.7±2.3	59.9±3.1	61.9±1.7	64.3±12.1	57.8±14.0
Gender						
Male	4 (50.0)	4 (50.0)	14 (56.0)	27 (55.1)	74 (63.2)	59 (52.2)
Female	4 (50.0)	4 (50.0)	11 (44.0)	22 (44.9)	43 (36.8)	54 (47.8)
Smoking status						
Non-smoker	5 (62.5)	5 (62.5)	15 (60.0)	23 (46.9)	48 (41.0)	69 (61.1)^*^
Smoker	3 (37.5)	3 (37.5)	10 (40.0)	26 (53.1)	69 (59.0)	44 (38.9)
Alcohol drinking						
Non-drinker	6 (75.0)	4 (50.0)	18 (72.0)	24 (49.0)	69 (59.0)	68 (60.2)
Drinker	2 (25.0)	4 (50.0)	7 (28.0)	25 (51.0)	48 (41.0)	45 (39.8)
Family history of cancer						
No	6 (75.0)	6 (75.0)	17 (68.0)	42 (85.7)	80 (68.4)	76 (67.3)
Yes	2 (25.0)	2 (25.0)	8 (32.0)	7 (14.3)	37 (31.6)	37 (32.7)
Histological type						
ADC	6 (75.0)		22 (88.0)		79 (67.5)	
SCC	2 (25.0)		3 (12.0)		22 (18.8)	
US-NSCLC	–		–		16 (13.7)	
TNM stage						
I	1 (12.5)		1 (4.0)		9 (7.7)	
II	–		1 (4.0)		5 (4.3)	
III	–		–		13 (11.1)	
IV	7 (87.5)		23 (92.0)		90 (76.9)	

^*^P value < 0.05. ADC, adenocarcinoma; HPs, healthy persons; NSCLC, non-small cell lung cancer; OLs, other lung diseases; SCC, squamous cell carcinoma; SD, standard deviation; US-NSCLC, unspecified non-small cell lung cancer.

**Table 2:** Differentially expressed miRNAs by miRNA profiling in the serum of patients with NSCLC *vs*. control groups

miRNAs	NSCLC *vs.* all controls			NSCLC *vs.* OLs			NSCLC *vs.* HPs		
	Differential expression	Fold change	*P*-value	Differential expression	Fold change	*P*-value	Differential expression	Fold change	*P*-value
MiR-769	−12.630	6.34 × 10^3^	0.005	−16.653	1.03 × 10^5^	0.007	−8.606	3.90 × 10^2^	0.062
MiR-339-3p	−10.242	1.21 × 10^3^	0.009	−10.597	1.55 × 10^3^	0.017	−9.887	9.47 × 10^2^	0.062
MiR-339-5p	−7.935	2.45 × 10^2^	0.021	−6.588	9.62 × 10^1^	0.089	−9.283	6.23 × 10^2^	0.042
MiR-519a	−5.338	4.05 × 10^1^	0.093	−3.303	9.87 × 10	0.734	−7.374	1.66 × 10^2^	0.017
MiR-1238	6.210	1.35 × 10^−2^	0.036	4.130	5.71 × 10^−2^	0.234	8.289	3.20 × 10^−3^	0.027
MiR-99a#	6.341	1.23 × 10^−2^	0.036	4.087	5.89 × 10^−2^	0.234	8.596	2.58 × 10^−3^	0.027
MiR-134	6.711	9.54 × 10^−3^	0.059	10.302	7.92 × 10^−4^	0.027	3.121	1.15 × 10^−1^	0.396
MiR-604	6.722	9.47 × 10^−3^	0.059	12.594	1.62 × 10^−4^	0.011	0.851	5.55 × 10^−1^	0.610
MiR-539	7.976	3.97 × 10^−3^	0.059	11.786	2.83 × 10^−4^	0.042	4.166	5.57 × 10^−2^	0.308
MiR-342	10.047	9.46 × 10^−4^	0.027	9.930	1.02 × 10^−3^	0.089	10.163	8.72 × 10^−4^	0.062

HPs, healthy persons; NSCLC, non-small cell lung cancer; OLs, other lung diseases.

**Table 3:** Predicted target genes for miR-339-3p and their pathways based on miRTarBase database

No	miRTarBase ID	Gene name (symbol)	Pathways
1	MIRT734201	BCL2 family apoptosis regulator (*MCL1*)	PI3K-Akt signaling pathway
2	MIRT733783	Forkhead box O1 (*FOXO1*)	FoxO signaling pathway, AMPK signaling pathway, insulin signaling pathway, thyroid hormone signaling pathway, insulin resistance, transcriptional misregulation in cancer, etc.
3	MIRT733784	Nuclear factor kappa B subunit 1 (*NFKB1*)	MAPK signaling pathway, Ras signaling pathway, cAMP signaling pathway, chemokine signaling pathway, NF-kappa B signaling pathway, HIF-1 signaling pathway, PI3K-Akt signaling pathway, apoptosis, toll-like receptor signaling pathway, NOD-like receptor signaling pathway, TNF signaling pathway, transcriptional misregulation in cancer, etc.
4	MIRT038179	Ribosomal protein L10a (*RPL10A*)	Ribosome
5	MIRT038180	ATP synthase, H+ transporting, mitochondrial F1 complex, beta polypeptide (*ATP5B*)	Oxidative phosphorylation, metabolic pathways, etc.
6	MIRT493527	Insulin-like growth factor 2 (*IGF2*)	Proteoglycans in cancer
7	MIRT495077	Hairy/enhancer-of-split related with YRPW motif-like (*HEYL*)	No data
8	MIRT496655	PITPNM family member 3 (*PITPNM3*)	No data
9	MIRT549396	Akirin 1 (*AKIRIN1*)	No data
10	MIRT562075	Kelch-like 15 (*KLHL15*)	No data
11	MIRT700815	Pleckstrin homology-like domain, family A, member 2 (*PHLDA2*)	No data
12	MIRT741362	Mex-3 RNA-binding family member D (*MEX3D*)	No data
13	MIRT741363	Short stature homeobox 2 (*SHOX2*)	No data
14	MIRT741364	WD and tetratricopeptide repeats 1 (*WDTC1*)	No data

**Table 4:** Logistic regression analysis of predictors associated with non-small cell lung cancer adenocarcinoma

Variables	NSCLC (*n* = 117) *vs.* OLs (*n* = 113)			ADC (*n* = 79) *vs.* OLs (*n* = 113)	
	Crude OR (95% CI)	Adjusted OR (95% CI)	*P*-value	Adjusted OR (95% CI)	*P*-value
Age < 60 *vs.* ≥ 60 years	2.27 (1.33–3.88)	2.08 (1.18–3.66)	0.011	1.94 (1.03–3.65)	0.04
Male *vs*. female	0.63 (0.37–1.08)				
Smoker *vs*. non-smoker	2.25 (1.33–3.82)	3.25 (1.54–6.85)	0.002	2.94 (1.31–6.59)	0.009
Drinker *vs*. non-drinker	1.05 (0.62–1.78)	0.52 (0.24–1.11)	0.093	0.49 (0.21–1.11)	0.087
Yes *vs*. No	0.95 (0.55–1.65)				
MiR-339-3p expression					
Low *vs*. High	2.62 (1.53–4.48)	2.43 (1.39–4.25)	0.002	2.96 (1.57–5.58)	<0.001

CI, confidence interval; NSCLC, non-small cell lung cancer; OLs, other lung diseases; OR, odds ratio.
